# Supplementary material for: A standardized image processing and data quality platform for rodent fMRI
Source: Nat Commun. 2024 Aug 7;15:6708. doi: 10.1038/s41467-024-50826-8 (PMC11306392; doi:10.1038/s41467-024-50826-8)
Supplement: Supplementary file 3 — Reporting Summary [file 41467_2024_50826_MOESM3_ESM.pdf]

nature portfolio

Corresponding author(s): Gabriel Desrosiers-Gregoire
Mallar Chakravarty

Last updated by author(s): May 21, 2024

Reporting Summary

Nature Portfolio wishes to improve the reproducibility of the work that we publish. This form provides structure for consistency and transparency in reporting. For further information on Nature Portfolio policies, see our Editorial Policies and the Editorial Policy Checklist.

Statistics

For all statistical analyses, confirm that the following items are present in the figure legend, table legend, main text, or Methods section.

n/a	Confirmed	
☐	☒	The exact sample size (*n*) for each experimental group/condition, given as a discrete number and unit of measurement
☐	☒	A statement on whether measurements were taken from distinct samples or whether the same sample was measured repeatedly
☒	☐	The statistical test(s) used AND whether they are one- or two-sided<br>*Only common tests should be described solely by name; describe more complex techniques in the Methods section.*
☒	☐	A description of all covariates tested
☒	☐	A description of any assumptions or corrections, such as tests of normality and adjustment for multiple comparisons
☐	☒	A full description of the statistical parameters including central tendency (e.g. means) or other basic estimates (e.g. regression coefficient) AND variation (e.g. standard deviation) or associated estimates of uncertainty (e.g. confidence intervals)
☒	☐	For null hypothesis testing, the test statistic (e.g. *F*, *t*, *r*) with confidence intervals, effect sizes, degrees of freedom and *P* value noted<br>*Give P values as exact values whenever suitable.*
☒	☐	For Bayesian analysis, information on the choice of priors and Markov chain Monte Carlo settings
☒	☐	For hierarchical and complex designs, identification of the appropriate level for tests and full reporting of outcomes
☐	☒	Estimates of effect sizes (e.g. Cohen's *d*, Pearson's *r*), indicating how they were calculated

Our web collection on statistics for biologists contains articles on many of the points above.

Software and code

Policy information about availability of computer code

Data collection	No data collection was conducted.
Data analysis	All code related to the RABIES software is openly accessible on Github (https://github.com/CoBrALab/RABIES). Installation and version control for all dependencies is managed through Docker containers, all readily available on Github container registry for download (https://github.com/CoBrALab/RABIES/pkgs/container/rabies). In this work, preprocessing was conducted with version 0.3.3 for datasets with anatomical scans and 0.4.7 for preprocessing using only EPI (for results in sup. table 2). For all results involving connectivity analyses, the pipeline steps for confound correction and analysis were conducted with version 0.5.0, while using the pre-generated preprocessing outputs from version 0.4.7. Of note, the same core registration algorithms are preserved across all software versions, and thus the same preprocessing outcome is expected. The software is predominantly written using python, and uses several packages, including NumPy v1.20.1, SciPy v1.6.2, NiPype v1.6.0, SimpleITK v2.0.2, NiBabel v3.2.1, Nilearn v0.7.1, pandas v1.2.4 and PyBIDS v0.13, as well as a modified version of ICA-AROMA (methods section 6). Additional software dependencies used within the RABIES software include ANTs58 v2.5.0, minc-toolkit v1.9.18, FSL59 (using MELODIC version 3.14) and AFNI60 23.1.09. Instructions and associated scripts for reproducing the RABIES outputs used in this paper can be found in a separate online repository (https://github.com/Gab-D-G/RABIES_paper_repro)61. The repository also regroups custom code for generating figures in this manuscript, which was done with a python environment regrouping the python packages listed above together with the RABIES python package.

For manuscripts utilizing custom algorithms or software that are central to the research but not yet described in published literature, software must be made available to editors and reviewers. We strongly encourage code deposition in a community repository (e.g. GitHub). See the Nature Portfolio guidelines for submitting code & software for further information.

Data

Policy information about availability of data

All manuscripts must include a data availability statement. This statement should provide the following information, where applicable:

- Accession codes, unique identifiers, or web links for publicly available datasets
- A description of any restrictions on data availability
- For clinical datasets or third party data, please ensure that the statement adheres to our policy

All data used for this work is publicly available through the OpenNeuro data sharing platform (with the exception of the NITRC rat, provided through the NITRC platform). The online link to each dataset is found in sup. table 2. Additionally, supplementary files which accompany the results presented here are accessible at this online repository https://doi.org/10.17605/OSF.IO/GT7EX. Source data is also provided for figures 3 and 5, and supplementary figures 15b, 16, 18 and 19.

Research involving human participants, their data, or biological material

Policy information about studies with human participants or human data. See also policy information about sex, gender (identity/presentation), and sexual orientation and race, ethnicity and racism.

Reporting on sex and gender	N/A
Reporting on race, ethnicity, or other socially relevant groupings	N/A
Population characteristics	N/A
Recruitment	N/A
Ethics oversight	N/A

Note that full information on the approval of the study protocol must also be provided in the manuscript.

Field-specific reporting

Please select the one below that is the best fit for your research. If you are not sure, read the appropriate sections before making your selection.

☒ Life sciences ☐ Behavioural & social sciences ☐ Ecological, evolutionary & environmental sciences

For a reference copy of the document with all sections, see nature.com/documents/nr-reporting-summary-flat.pdf

Life sciences study design

All studies must disclose on these points even when the disclosure is negative.

Sample size	The preprocessing quality control results in part 1 of the manuscript included a total of 318 mice with 440 functional scans, and 166 rats with 233 functional scans across datasets. Analyses in part 2 of the manuscript include a total of 273 mice with 367 fMRI scans. No calculations were performed to estimate sample size. In this study, instead of aiming for a specific sample size, we gathered all publicly available mouse and rat fMRI data which we could access at the beginning the study to establish a representative survey of data acquisitions in the field.
Data exclusions	All data was included for evaluating preprocessing. For subsequent connectivity analyses, only the REST-AWK and the mouse multicenter datasets were included. Other mouse datasets were excluded to avoid including multiple datasets acquired from the same site. Rats datasets were not included in our comparison of connectivity quality between sites, as we only gathered 3 sites from that species, making it insufficient for conducting proper multi-site comparisons. 2 additional scans were excluded for failing preprocessing quality control.
Replication	Replication is explicitly built in as we seek to find a generalizable set of rules across the data from the different sites used.
Randomization	Experiments were conducted both at the level of individual MRI scans (where no grouping is considered) and at the level of acquisition sites. In the later case, experimental grouping was retrospective, and thus randomization was likely unnecessary. There were no relevant covariate to account for in our comparison between acquisition sites.
Blinding	No blinding was conducted for the manual inspection of the RABIES data quality reports. We did not consider this necessary, as we did not have prior hypotheses and the inspector did not have affiliations to a particular dataset, which could have been of concern for biases. Also, no statistical tests were conducted for inspecting group differences - the affiliation of datasets to certain data quality categories was descriptive.

Reporting for specific materials, systems and methods

We require information from authors about some types of materials, experimental systems and methods used in many studies. Here, indicate whether each material, system or method listed is relevant to your study. If you are not sure if a list item applies to your research, read the appropriate section before selecting a response.

Materials & experimental systems

n/a	Involved in the study
☒	☐ Antibodies
☒	☐ Eukaryotic cell lines
☒	☐ Palaeontology and archaeology
☐	☒ Animals and other organisms
☒	☐ Clinical data
☒	☐ Dual use research of concern
☒	☐ Plants

Methods

n/a	Involved in the study
☒	☐ ChIP-seq
☒	☐ Flow cytometry
☐	☒ MRI-based neuroimaging

Animals and other research organisms

Policy information about studies involving animals; ARRIVE guidelines recommended for reporting animal research, and Sex and Gender in Research

Laboratory animals	C57Bl/6J mouse strain with both male and female. Rat strains include Wistar (both male and females), Long-Evans (male only), and Sprague-Dawley (male and females).
Wild animals	Did not involve wild animals.
Reporting on sex	When combining the different datasets, there was a mix of both sexes for both mice and rat datasets (see above). We did not carry sex-specific analyses, since for the main datasets we analyzed (https://openneuro.org/datasets/ds001720/versions/1.0.2 and https://openneuro.org/datasets/ds001653/versions/1.0.2) sex the information was incomplete or only a single sex was included per dataset.
Field-collected samples	No field samples collected.
Ethics oversight	Provided along original submission of the online databases.

Note that full information on the approval of the study protocol must also be provided in the manuscript.

Plants

Seed stocks	*Report on the source of all seed stocks or other plant material used. If applicable, state the seed stock centre and catalogue number. If plant specimens were collected from the field, describe the collection location, date and sampling procedures.*
Novel plant genotypes	*Describe the methods by which all novel plant genotypes were produced. This includes those generated by transgenic approaches, gene editing, chemical/radiation-based mutagenesis and hybridization. For transgenic lines, describe the transformation method, the number of independent lines analyzed and the generation upon which experiments were performed. For gene-edited lines, describe the editor used, the endogenous sequence targeted for editing, the targeting guide RNA sequence (if applicable) and how the editor was applied.*
Authentication	*Describe any authentication procedures for each seed stock used or novel genotype generated. Describe any experiments used to assess the effect of a mutation and, where applicable, how potential secondary effects (e.g. second site T-DNA insertions, mosiacism, off-target gene editing) were examined.*

Magnetic resonance imaging

Experimental design

Design type	Resting-state
Design specifications	*Specify the number of blocks, trials or experimental units per session and/or subject, and specify the length of each trial or block (if trials are blocked) and interval between trials.*
Behavioral performance measures	*State number and/or type of variables recorded (e.g. correct button press, response time) and what statistics were used to establish that the subjects were performing the task as expected (e.g. mean, range, and/or standard deviation across subjects).*

Acquisition

Imaging type(s)	functional
Field strength	4.7T, 7T,9.4T,11.7T
Sequence & imaging parameters	Gradient-echo EPI sequence. Other imaging parameters vary between dataset, and details can be found in the original dataset repository.
Area of acquisition	At minimum including most of the cortex, but variable with regards to the inclusion of olfactory bulbs and cerebellum.

Diffusion MRI ☐ Used ☒ Not used

Preprocessing

Preprocessing software	RABIES version 0.3.3, 0.4.7 and 0.5.0 (see code reproducibility instructions for details)
Normalization	The normalization was non-linear, using structural scans when available in part 1 of the manuscript, or using the EPI otherwise. For part 2, the EPI was always used for normalization.
Normalization template	DSURQE ex-viso atlas for mice, Fischer 344 atlas for rats.
Noise and artifact removal	Variable, as this was a topic of study in the manuscript.
Volume censoring	Censoring based on framewise displacement and/or DVARS threshold (topic of investigation in the manuscript)

Statistical modeling & inference

Model type and settings	N/A
Effect(s) tested	N/A

Specify type of analysis: ☐ Whole brain ☐ ROI-based ☒ Both

Anatomical location(s)	Seeds were manually drawn in regions of interest corresponding to the anterior cingulate or the right primary somatosensory cortex.
Statistic type for inference (See Eklund et al. 2016)	N/A
Correction	N/A

Models & analysis

n/a	Involved in the study
☐	☒ Functional and/or effective connectivity
☒	☐ Graph analysis
☐	☒ Multivariate modeling or predictive analysis

Functional and/or effective connectivity	Using pearson correlation.
Multivariate modeling and predictive analysis	Group independent component analysis (ICA) and dual regression.
